# Breast cancer susceptibility loci and mammographic density

**DOI:** 10.1186/bcr2127

**Published:** 2008-08-05

**Authors:** Rulla M Tamimi, David Cox, Peter Kraft, Graham A Colditz, Susan E Hankinson, David J Hunter

**Affiliations:** 1 Channing Laboratory, Department of Medicine, Brigham and Women's Hospital and Harvard Medical School, 181 Longwood Avenue, Boston, MA, 02115, USA; 2 Department of Epidemiology, Harvard School of Public Health, 677 Huntington Avenue, Boston, MA, 02115, USA; 3 Department of Surgery, Washington University School of Medicine, 660 S. Euclid Avenue, St. Louis, MO 63110, USA

## Abstract

**Introduction:**

Recently, the Breast Cancer Association Consortium (BCAC) conducted a multi-stage genome-wide association study and identified 11 single nucleotide polymorphisms (SNPs) associated with breast cancer risk. Given the high degree of heritability of mammographic density and its strong association with breast cancer, it was hypothesised that breast cancer susceptibility loci may also be associated with breast density and provide insight into the biology of breast density and how it influences breast cancer risk.

**Methods:**

We conducted an analysis in the Nurses' Health Study (n = 1121) to assess the relation between 11 breast cancer susceptibility loci and mammographic density. At the time of their mammogram, 217 women were premenopausal and 904 women were postmenopausal. We used generalised linear models adjusted for covariates to determine the mean percentage of breast density according to genotype.

**Results:**

Overall, no association between the 11 breast cancer susceptibility loci and mammographic density was seen. Among the premenopausal women, three SNPs (rs12443621 [TNRc9/LOC643714], rs3817198 [lymphocyte-specific protein-1] and rs4666451) were marginally associated with mammographic density (p < 0.10). All three of these SNPs showed an association that was consistent with the direction in which these alleles influence breast cancer risk. The difference in mean percentage mammographic density comparing homozygous wildtypes to homozygous variants ranged from 6.3 to 8.0%. None of the 11 breast cancer loci were associated with postmenopausal breast density.

**Conclusion:**

Overall, breast cancer susceptibility loci identified through a genome-wide association study do not appear to be associated with breast cancer risk.

## Introduction

Mammographic density is one of the strongest risk factors for breast cancer. Women with 75% or more breast density are at a four- to six-fold greater risk of breast cancer than women with no density [1,2]. The mechanism by which mammographic density increases breast cancer risk is unclear.

Results from twin studies have estimated that inherited genetic influences account for 60 to 67% of the variation in mammographic density [3]. Given its high degree of heritability [3,4], a number of studies have examined genetic variation and mammographic density utilising a candidate gene approach focusing primarily on polymorphisms in oestrogen synthesis and metabolising genes with inconclusive results [5-10]. In addition, polymorphisms in growth factor genes – such as insulin-like growth factor (IGF)-1 [11], IGF binding protein 3 [12] and pituitary growth hormone [13] – have been reported to be associated with mammographic density, although these findings have not yet been examined or replicated in independent studies.

Recently, the Breast Cancer Association Consortium (BCAC) conducted a multi-stage genome-wide association study of breast cancer cases and controls and identified 11 single nucleotide polymorphisms (SNPs) associated with risk [14]. The Nurses' Health Study breast cancer nested case-control study contributed to the third stage of this genome-wide association study. Given the strong association between mammographic density and breast cancer, breast cancer susceptibility loci may also be associated with breast density and provide insight into the biology of breast density and how it influences breast cancer risk. We conducted a cross-sectional study in the Nurses' Health Study (n = 1121) to assess the relation between breast cancer susceptibility loci identified from a multi-stage genome-wide association study and mammographic density.

## Materials and methods

The Nurses' Health Study was initiated in 1976, with 121,700 US-registered nurses age 30 to 55 years returning an initial questionnaire [15]. Information on body mass index (BMI), reproductive history, age at menopause and postmenopausal hormone use, as well as diagnosis of cancer and other diseases are updated every two years through questionnaires. During 1989 and 1990, blood samples were collected from 32,826 women [16]. In general, blood samples were returned within 26 hours of being drawn and were immediately centrifuged, aliquoted into plasma, red blood cells and buffy coat fractions, and stored in liquid nitrogen freezers. The follow-up rate among women who provided blood samples was 99% through 1998.

We conducted a cross-sectional analysis among controls from a breast cancer case-control study nested within the Nurses' Health Study cohort. This nested case-control study included breast cancer cases diagnosed after blood collection but before 1 June 1998 and matched controls [17]. The collection of film mammograms was targeted to breast cancer cases and matched controls through the 1998 follow-up cycle. We collected mammograms taken as close as possible to the date of blood collection (1989 to 1990). This collection has been described in detail in a previous publication [11].

This study was approved by the Committee on the Use of Human Subjects in Research at Brigham and Women's Hospital.

To assess mammographic density, the craniocaudal views of both breasts were digitised at 261 microns/pixel with a Lumysis 85 laser film scanner, which covers a range of 0 to 4.0 optical density. The software for computer-assisted thresholding was developed at the University of Toronto [18] and this measure of mammographic breast density was highly reproducible within this study [19]. We used the average percentage density of both breasts for this analysis. We also evaluated the association of these SNPs with the absolute area of mammographic density, but because results were similar and percentage breast density has been a stronger predictor of breast cancer risk than area of dense tissue in many [1,20-23] but not all [24] studies, we present the results for percentage mammographic density as our primary analyses and include the association with dense and non-dense area in supplementary tables.

As part of the third stage of the BCAC multi-stage genome-wide association study, 30 SNPs were genotyped in the Nurses' Health Study breast cancer nested case-control samples. Although genotype data are available for all 30 SNPs, we present results for the 11 SNPs reported to be associated with breast cancer after all three stages as our primary results. Analyses examining the association between the other 19 SNPs and breast density were considered secondary (see supplementary table 1 in additional data file 1).

**Table 1:** Descriptive characteristics of study population (n = 1121) at the time of mammography, Nurses' Health Study (1989–1998)

Characteristic		
*Mean values*	Mean	SD
Age, y	58.4	7.3
Body mass index, kg/m^2^	25.6	4.6
Percentage mammographic density, %	27.1	20.6
		
*Frequencies*^a^	N	%
Menopausal/hormone status		
Premenopausal	217	19.4
Postmenopausal/never user	371	33.1
Postmenopausal/past user	317	28.3
Postmenopausal/current user	216	19.3
Alcohol consumption		
None	327	31.8
<5 g/day	346	33.6
5 to 14.9 g/day	221	21.5
15+ g/day	135	13.1
Parity/age at first birth		
Nulliparous	83	7.5
Parous/<25 years	549	49.6
Parous/25 to 29 years	382	34.5
Parous/>30	93	8.4
Prior benign breast disease	465	41.5
Family history of breast cancer	135	12.0

^a ^Numbers may not be added to total due to missing data.

Genotyping was conducted using a fluorescent 5' endonuclease assay and the ABI-PRISM 7900 (Taqman) for sequence detection. For quality control, about 10% of samples were included as blinded duplicates in the genotyping runs. The concordance for replicate samples was more than 99%.

We used generalised linear models adjusted for covariates to determine the mean percentage breast density according to genotype. To determine if there was a linear trend with increasing variant alleles, we calculated p values from Wald statistics including an ordinal variable for genotype regressed on square root transformed percentage mammographic density. Covariate information at the time of the mammogram was assessed using data from biennial questionnaires completed before the date of the mammogram. Although it is unlikely that these factors are confounders of the genotype and mammographic density relationship, these variables do explain the substantial variation in the outcome. In addition, genetic variants may be associated with mammographic density through its association with other covariates, such as BMI. Because our goal was to determine if breast cancer susceptibility loci are associated with mammographic density independent of factors know to influence breast density, we included known predictors of mammographic density in multivariate models.

Percentage mammographic density is considerably lower in postmenopausal women than in premenopausal women. It has been suggested that premenopausal breast density may be more highly heritable than postmenopausal density [25], and that different genes may be associated with premenopausal density rather than with postmenopausal density [26]. We therefore *a priori *considered that genes may differentially be associated with premenopausal and postmenopausal mammographic density. Because there was evidence that the association between some of the breast cancer susceptibility loci and breast density varied according to menopausal status at mammogram we included results stratified by menopausal status. Data analysis was conducted using SAS statistical software version 9.1. All p values presented are two-sided tests of statistical significance.

## Results

This study examined the association between 11 breast cancer susceptibility loci and mammographic density in 1121 women in the Nurses' Health Study. The mean age of participants at the time of mammography was 58.4 years (Table 1). At the time of their mammogram, 217 women were premenopausal, with a mean (SD) age at mammography of 49.1 (3.3) years. Among premenopausal women, the mean percentage mammographic density was 39.7 (22.2)% (range 0.6 to 90.3%). At the time of their mammogram, 904 women were postmenopausal with a mean age at mammography of 60.6 (6.2) years. Among postmenopausal women, the mean percentage mammographic density was 24.1 (19.0)% (range 0.0 to 86.5%). Of the postmenopausal women, 41% had never used postmenopausal hormones, 24% were currently using postmenopausal hormones and 35% were former users at the time of their mammograms. European ancestry was self-reported by 98.8% of the women in the study.

Overall, there was no significant association between the 11 breast cancer susceptibility loci and mammographic density (Table 2). Of the 11 breast cancer susceptibility loci, three were associated with premenopausal mammographic density at p < 0.10 (Table 3). The variant allele of rs12443621 (TNRc9/LOC643714) was positively associated with mammographic density; the mean percentage mammographic density among homozygous variants (42.5%) was 6.3% percentage points greater than those homozygous for the wildtype allele (36.2%). This association was primarily due to the association with dense area (p = 0.04; see supplementary Table 2 in additional file 2), rather than non-dense area (p = 0.30; see supplementary Table 3 in additional file 3). The variant allele of rs3817198 (lymphocyte-specific protein-1 [LSP1]) was positively associated with mammographic density; the mean percentage mammographic density among homozygous variants (41.0%) was 7.5% percentage points greater than those homozygous for the wildtype allele (33.5%). This association also appears to be due to the association with dense area (p = 0.04; see supplementary Table 2 in additional file 2), rather than with non-dense area (p = 0.15; see supplementary Table 3 in additional file 3). The variant allele of rs4666451 was inversely associated with mammographic density; the mean percentage mammographic density among homozygous variants (33.6%) was 8.0% percentage points lower than those homozygous for the wildtype allele (41.6%). In contrast, this association was primarily due to the association with non-dense area (p-trend = 0.02; see supplementary Table 3 in additional file 3), rather than with dense area (p-trend = 0.22; see supplementary Table 2 in additional file 2). None of the 11 breast cancer loci were associated with postmenopausal breast density (Table 3). We also conducted secondary analyses among postmenopausal women restricted to women who were not taking postmenopausal hormones at the time of mammography (n = 688). In general, these results were consistent with results among all postmenopausal women (data not shown).

**Table 2:** Mean percentage of mammographic density (MD) according to breast cancer susceptibility loci, Nurses' Health Study controls (1989 to 1998)

		N	Mean %MD^a^	Mean %MD^b^
**rs2981582**	G/G	421	26.9	26.7
	G/A	539	26.9	26.6
	A/A	160	26.8	28.0
p value ^d^			0.56	0.98
**rs12443621**	A/A	298	25.0	25.7
	A/G	561	27.3	26.9
	G/G	267	27.4	27.1
p value ^d^			0.12	0.25
**rs13281615**	A/A	377	27.6	27.2
	A/G	553	25.9	26.3
	G/G	198	28.1	27.5
p value ^d^			0.90	0.91
**rs3817198**	A/A	489	26.0	25.9
	A/G	491	27.8	27.8
	G/G	119	25.9	26.1
p value ^d^			0.95	0.79
**rs889312**	T/T	622	28.3	28.2
	T/G	428	24.7	25.1
	G/G	76	28.6	27.3
p value ^d^			0.12	0.06
**rs4666451**	G/G	405	26.9	27.4
	A/G	531	27.2	26.7
	A/A	184	25.7	25.8
p value ^d^			0.43	0.13
**rs2107425**	G/G	572	27.5	27.5
	G/A	440	25.8	25.8
	A/A	108	26.7	26.3
p value ^d^			0.39	0.24
**rs981782**	A/A	336	27.9	27.0
	A/C	544	26.5	26.9
	C/C	231	26.7	26.8
p value ^d^			0.15	0.40
**rs8051542**	G/G	307	26.8	26.8
	G/A	383	25.7	25.6
	A/A	173	29.3	29.2
p value ^d^			0.67	0.64
**rs30099**	C/C	929	26.9	27.0
	C/T	193	26.0	25.5
	T/T	8	28.0	24.0
p value ^d^			0.81	0.28
**rs3803662**	G/G	590	26.2	26.4
	G/A	434	27.5	26.9
	A/A	90	27.1	28.6
p value ^d^			0.53	0.37

^a^Age adjusted.

^b^Multivariate adjusted for the following: age (continuous), body mass index (BMI) (continuous), alcohol consumption (none, <5 g/day, 5 to 14.9 g/day, 15+ g/day), age at first birth/parity (nulliparous, age at first birth <25, age at first birth 25 to 29, age at first birth >30), history of benign breast disease (yes/no), family history of breast cancer (yes/no).

^c^Multivariate adjusted for the following: age, BMI, alcohol consumption, age at first birth/parity, history of benign breast disease, family history of breast cancer, postmenopausal status/hormone use (premenopausal, never user, current user, past user).

^d^p value based on genotype coded as ordinal variable regressed on square root transformed MD.

**Table 3:** Mean percentage mammographic density (MD) according to breast cancer susceptibility loci, Nurses' Health Study controls (1989–1998)

		Premenopausal (n = 217)			Postmenopausal (n = 904)		
		N	Mean %MD^a^	Mean %MD^b^	N	Mean %MD^a^	Mean %MD^c^

**rs2981582**	G/G	73	37.4	37.9	332	24.4	24.2
	G/A	112	40.5	40.7	407	23.9	23.6
	A/A	28	41.7	38.4	131	23.7	25.3
p value ^d^			0.27	0.62		0.34	0.82
**rs12443621**	A/A	51	36.0	**36.2**	236	22.9	23.4
	A/G	104	38.9	**39.1**	442	24.5	24.2
	G/G	54	43.7	**42.5**	202	23.5	23.8
p value ^d^			0.05	**0.06**		0.70	0.72
**rs13281615**	A/A	70	39.7	38.2	290	25.2	25.0
	A/G	94	36.0	38.2	445	23.7	23.9
	G/G	47	45.8	43.1	144	22.9	22.8
p value ^d^			0.45	0.52		0.36	0.32
**rs3817198**	A/A	80	33.9	**33.5**	396	24.2	24.0
	A/G	106	42.6	**42.3**	368	24.4	24.6
	G/G	20	39.5	**41.0**	93	22.7	22.8
p value ^d^			0.04	**0.01**		0.39	0.56
**rs889312**	T/T	107	41.1	41.3	499	25.6	25.4
	T/G	89	38.5	38.2	321	21.5	22.1
	G/G	14	38.8	37.2	58	26.8	25.3
p value ^d^			0.40	0.21		0.17	0.14
**rs4666451**	G/G	74	40.7	**41.6**	314	24.0	24.5
	A/G	96	40.6	**39.5**	418	24.2	23.8
	A/A	36	33.0	**33.6**	144	24.3	24.3
p value ^d^			0.13	**0.06**		0.90	0.40
**rs2107425**	G/G	105	41.7	40.7	449	24.3	24.6
	G/A	87	36.8	37.2	337	23.4	23.3
	A/A	18	37.5	39.6	85	24.6	23.6
p value ^d^			0.11	0.29		0.79	0.37
**rs981782**	A/A	64	37.7	37.5	258	25.5	24.6
	A/C	100	39.4	39.2	429	23.7	24.1
	C/C	47	42.3	42.5	175	23.2	23.4
p value ^d^			0.39	0.27		0.08	0.22
**rs8051542**	G/G	63	41.0	40.5	234	23.2	23.4
	G/A	73	40.0	40.1	296	22.3	22.3
	A/A	32	37.9	37.8	137	27.6	27.3
p value ^d^			0.58	0.65		0.36	0.43
**rs30099**	C/C	175	38.8	38.8	723	24.4	24.5
	C/T	34	40.5	39.9	151	22.6	22.4
	T/T	0			8	26.0	21.8
p value ^d^			0.45	0.51		0.49	0.16
**rs3803662**	G/G	107	37.0	36.9	458	23.9	24.1
	G/A	77	43.9	42.7	344	24.0	23.4
	A/A	20	32.7	36.1	69	26.5	28.0
p value ^d^			0.55	0.34		0.64	0.50

^a^Age adjusted.

^b^Multivariate adjusted for the following: age (continuous), body mass index (BMI) (continuous), alcohol consumption (none, <5 g/day, 5 to 14.9 g/day, 15+ g/day), age at first birth/parity (nulliparous, age at first birth <25, age at first birth 25 to 29, age at first birth >30), history of benign breast disease (yes/no), family history of breast cancer (yes/no).

^c^Multivariate adjusted for the following: age, BMI, alcohol consumption, age at first birth/parity, history of benign breast disease, family history of breast cancer, postmenopausal hormone use (never user, current user, past user).

^d^p value based on genotype coded as ordinal variable regressed on square root transformed MD.

In a secondary analysis, we examined the 19 additional SNPs that were genotyped in this study population as part of stage 3 of the genome-wide association study (see supplementary Table 1 in additional file 1) but were reported to not be significantly associated with breast cancer after the final stage. Two of these SNPs (rs6843340 and rs2298075) were associated with mammographic density in the population overall. rs6843340 was associated with both premenopausal and postmenopausal density, while rs2298075 was associated with only postmenopausal density (see supplementary Table 4 in additional file 4). In addition, rs10508468 was associated with premenopausal breast density and rs6469633 was associated with postmenopausal breast density only (see supplementary Table 4 in additional file 4).

## Discussion

In this study, we found that overall none of the breast cancer susceptibility loci were associated with mammographic density. However, some breast cancer susceptibility loci were associated with premenopausal breast density. We did not observe an association between any of these loci and postmenopausal breast density. These loci were originally identified from a three-stage genome-wide association study. The first stage of the genome-wide association study was among 390 breast cancer patients selected with a strong family history of breast cancer of at least two affected first-degree family relatives and 364 controls [14]. Women with a family history are more likely to develop breast cancer at an early age. It is known that some breast cancer risk factors are differentially associated with premenopausal and postmenopausal breast cancer [27], hence it may be the case that the specific gene contribution to density may also vary by menopausal status. However, multiple lines of evidence suggest that the determinants of breast density may differ at different ages [26,28,29]. Thus, it is possible that different sets of genetic variants may be highly associated with premenopausal and postmenopausal breast density. If this is the case it may explain why these SNPs were associated with premenopausal breast density only and not postmenopausal breast density.

A limitation of the current study is the relatively small number of premenopausal women with density measurements (n = 217). Given the number of tests conducted, it is possible that these findings are the result of chance. However, the direction and strength of the association observed between the three polymorphisms and premenopausal mammographic density suggest that these genes may play an important role in determining premenopausal breast density. All three associated SNPs are consistent with the direction in which these alleles influence breast cancer risk [14]. The variant alleles of both rs12443621 and rs3817198 were associated with increasing breast density in this study and with increasing breast cancer risk in the BCAC (Odds ratio [OR] for rs12443621 homozygous variant vs homozygous wildtype = 1.23, 95% confidence interval [CI] 1.17 to 1.30 [14]; OR for rs3817198 homozygous variant vs homozygous wildtype = 1.17, 95%CI 1.08 to 1.25 [14]). The variant allele of rs4666451 was inversely associated with breast density and with a reduced risk of breast cancer in the genome-wide association study (OR homozygous variant vs homozygous wildtype = 0.93, 95%CI 0.87 to 0.99) [14]. In addition, the difference in mean percentage mammographic density comparing homozygous wildtypes to homozygous variants ranged from 6.3 to 8.0 percentage points. These differences in mammographic density are in the same range that has been associated previously with changes in mammographic density observed with exposure to postmenopausal hormones[30,31] and tamoxifen [32]. It is estimated that a 5% higher mammographic density is associated with a 7% increase in breast cancer risk [1]; thus, the estimated risk of breast cancer associated with these three SNPs, and attributable to their influence on mammographic density, would therefore range from 1.09 to 1.12.

Two of the three SNPs associated with premenopausal breast density are in known genes: TNRc9/LOC643714 (rs12443621) and LSP1. It is unclear what the function of the TNRc9 (also known as TOX3) gene is; however, it contains a putative high mobility group box motif which suggests that it may act as a transcription factor and this gene has been implicated in breast cancer metastasis to the bone [33]. Another variant in the TNRc9 gene (rs3803662) also emerged from an independent genome-wide association study of oestrogen-receptor positive breast cancer in an Icelandic population [34]. rs3817198 in the LSP1 gene (also known as WP43) is a cytoskeletal protein expressed in haematopoietic and endothelial cells. rs4666451 is located on 2p with no known gene function, although the region did appear in genome-wide linkage analyses of familial breast cancer [35]. It remains to be seen whether these SNPs exhibit their influence on breast cancer risk through mammographic density. Because these SNPs are associated with modest associations in breast cancer risk, larger studies with premenopausal density measurements on breast cancer cases and controls will be necessary to address this question.

SNPs in intron 2 of fibroblast growth factor receptor 2 (FGFR2) have emerged as top hits from multiple genome-wide association studies of breast cancer [14,36]. We did not detect an association between an FGFR2 (rs2981582) SNP and breast density in the current study. Homozygous variants of rs2981582 or other SNPs in high linkage disequilibrium are estimated to confer about a 60% increase in breast cancer risk relative to homozygous wildtypes [14,36]. Assuming the association between FGFR2 variants and breast cancer is mediated through breast density, one would expect to see a 33% difference in mammographic density comparing homozygous variants with homozygous wildtypes. The current study consisted of sufficiently high numbers of both premenopausal and postmenopausal women to detect such a difference. The lack of association of the SNP in FGFR2 and other breast cancer loci with breast density in this study suggests that some genes influence breast cancer risk independent of breast density.

Our primary analyses focused on the 11 SNPs identified after a multi-stage genome-wide association study with p ≤ 0.001 in the combined analysis. We also examined 20 additional SNPs that were either no longer significant at this cutoff or not consistent with the direction originally observed. There is suggestive evidence that three of these SNPs (rs2298075, rs684340 and rs6469633) are associated with breast density in a direction consistent with the observed association with breast cancer risk. The p value in the combined BCAC study for these three SNPs ranged from 0.002 to 0.13. Thus although they were not considered significantly associated with breast cancer at the defined cutoff, their borderline significance with breast cancer risk and consistent association with breast density warrant further investigation of these SNPs in relation to breast density and breast cancer risk.

To our knowledge, this is the first study to examine the association between these replicated breast cancer susceptibility loci and mammographic density in a cancer-free population. Lee and colleagues [37] genotyped six breast cancer susceptibility loci identified from genome-wide association studies among 516 young (<50 years of age) breast cancer patients. Five of these six SNPs were included in the present study. Overall, they observed no association between the SNPs and mammographic density. However, among women with oestrogen-receptor positive breast cancer rs3817198 was associated with mammographic density (p = 0.02). The direction of the association observed was consistent with our findings among premenopausal women.

The majority of the current study population was postmenopausal at the time of mammography and we were limited by the number of premenopausal women with breast density measurements. Given that the first stage of the genome-wide association study was among women with a strong family history, these SNPs may be more likely to be associated with premenopausal density. It remains to be seen whether other breast cancer susceptibility loci from other study designs are associated with postmenopausal breast density.

## Conclusion

Overall, breast cancer susceptibility loci identified through a genome-wide association study do not appear to be associated with breast cancer risk.

## Abbreviations

BCAC = Breast Cancer Association Consortium; BMI = Body mass index; CI = Confidence interval; FGFR2 = fibroblast growth factor receptor 2; LSP1 = lymphocyte-specific protein-1; OR = Odds ratio; SNP = Single nucleotide polymorphism.

## Competing interests

The authors declare that they have no competing interests.

## Authors' contributions

RMT was responsible for data analyses, manuscript preparation and editing. RMT, PK, GAC and DJH made substantial contributions to the study design and to the interpretation of data. DC and SEH contributed to interpretation of data and manuscript editing. All authors read and approved the final manuscript.

## Supplementary Material

Additional file 1 A Word document containing a table that lists the mean percentage of mammographic density according to second-stage single nucleotide polymorphisms (SNPs) that were not validated in stage 3 Breast Cancer Association Consortium SNPs among controls only, Nurses' Health Study controls (1989 to 1998). Click here for file

Additional file 2 A Word document containing a table that lists the mean absolute (cm^2^) dense area according to breast cancer susceptibility loci, Nurses' Health Study controls (1989 to 1998). Click here for file

Additional file 3 A Word document containing a table that lists the mean absolute (cm^2^) non-dense area according to breast cancer susceptibility loci, Nurses' Health Study controls (1989 to 1998). Click here for file

Additional file 4 A Word document containing a table that lists the mean percentage mammographic density according to second-stage SNPs that were not validated in stage 3 BCAC SNPs among controls. Click here for file
