# Supplementary material for: Breast cancer susceptibility loci and mammographic density
Source: Breast Cancer Res. 2008 Aug 5;10(4):R66. doi: 10.1186/bcr2127 (PMC2575539; doi:10.1186/bcr2127)
Supplement: Additional file 1 — A Word document containing a table that lists the mean percentage of mammographic density according to second-stage single nucleotide polymorphisms (SNPs) that were not validated in stage 3 Breast Cancer Association Consortium SNPs among controls only, Nurses' Health Study controls (1989 to 1998). [file bcr2127-S1.doc]

Supplementary Table 1: Mean percent mammographic density according to 2^nd^ stage SNPs that were not validated in Stage 3 BCAC SNPs among controls only, Nurses’ Health Study controls (1989-1998)

|  |  |  | N | Mean %MD^1^ | Mean %MD^2^ |  |
| --- | --- | --- | --- | --- | --- | --- |
| **rs3857481** | G/G |  | 527 | 25.9 | 25.8 |  |
|  | A/G |  | 495 | 27.8 | 27.8 |  |
|  | A/A |  | 106 | 25.4 | 25.2 |  |
| P-trend ^3^ |  |  |  | 0.69 | 0.60 |  |
| **rs1318703** | A/A |  | 393 | 26.8 | 26.9 |  |
|  | A/G |  | 537 | 26.3 | 26.4 |  |
|  | G/G |  | 185 | 27.2 | 26.4 |  |
| P-trend ^3^ |  |  |  | 0.91 | 0.74 |  |
| **rs16998733** | G/G |  | 886 | 26.8 | 26.8 |  |
|  | A/G |  | 216 | 27.9 | 27.7 |  |
|  | A/A |  | 20 | 20.1 | 21.4 |  |
| P-trend ^3^ |  |  |  | 0.77 | 0.70 |  |
| **rs2314099** | C/C |  | 998 | 26.9 | 26.8 |  |
|  | C/A |  | 121 | 25.6 | 25.7 |  |
|  | A/A |  | 6 | 27.4 | 33.3 |  |
| P-trend ^3^ |  |  |  | 0.52 | 0.77 |  |
| **rs4841365** | G/G |  | 541 | 27.4 | 27.2 |  |
|  | G/C |  | 454 | 26.5 | 26.7 |  |
|  | C/C |  | 107 | 26.3 | 26.0 |  |
| P-trend ^3^ |  |  |  | 0.63 | 0.69 |  |
| **rs7313833** | G/G |  | 505 | 26.7 | 26.7 |  |
|  | A/G |  | 483 | 27.1 | 27.1 |  |
|  | A/A |  | 131 | 28.3 | 28.2 |  |
| P-trend ^3^ |  |  |  | 0.50 | 0.40 |  |
| **rs11235127** | G/G |  | 756 | 26.9 | 26.7 |  |
|  | A/G |  | 314 | 26.9 | 27.1 |  |
|  | A/A |  | 40 | 24.6 | 25.7 |  |
| P-trend ^3^ |  |  |  | 0.90 | 0.66 |  |
| **rs17157070** | A/A |  | 1034 | 27.2 | 26.9 |  |
|  | A/G |  | 91 | 24.5 | 27.5 |  |
|  | G/G |  | 3 | - | 6.2 |  |
| P-trend ^3^ |  |  |  | 0.01 | 0.48 |  |
| **rs6469633** | A/A |  | 637 | 25.7 | 25.9 |  |
|  | A/G |  | 425 | 28.3 | 27.9 |  |
|  | G/G |  | 55 | 27.8 | 28.1 |  |
| P-trend ^3^ |  |  |  | 0.05 | 0.06 |  |
| **rs4331913** | G/G |  | 379 | 26.6 | 26.5 |  |
|  | G/A |  | 553 | 27.2 | 27.4 |  |
|  | A/A |  | 193 | 25.9 | 25.3 |  |
| P-trend ^3^ |  |  |  | 0.38 | 0.19 |  |
| **rs6843340** | G/G |  | 306 | 24.5 | **24.1** |  |
|  | G/A |  | 552 | 27.9 | **27.9** |  |
|  | A/A |  | 267 | 27.5 | **27.8** |  |
| P-trend ^3^ |  |  |  | 0.03 | **0.004** |  |
| **rs2049621** | C/C |  | 923 | 26.8 | 27.0 |  |
|  | C/G |  | 193 | 26.7 | 25.8 |  |
|  | G/G |  | 11 | 33.4 | 32.5 |  |
| P-trend ^3^ |  |  |  | 0.73 | 0.71 |  |
| **Rs10508468** | A/A |  | 456 | 26.5 | 25.9 |  |
|  | A/G |  | 511 | 27.0 | 27.8 |  |
|  | G/G |  | 154 | 27.2 | 26.1 |  |
| P-trend ^3^ |  |  |  | 0.65 | 0.42 |  |
| **rs13110927** | G/G |  | 288 | 26.2 | 26.5 |  |
|  | G/A |  | 573 | 27.5 | 27.1 |  |
|  | A/A |  | 262 | 25.6 | 25.9 |  |
| P-trend ^3^ |  |  |  | 0.80 | 0.75 |  |
| **rs4954956** | G/G |  | 620 | 26.7 | 26.6 |  |
|  | G/A |  | 434 | 27.1 | 27.1 |  |
|  | A/A |  | 78 | 28.3 | 29.0 |  |
| P-trend ^3^ |  |  |  | 0.53 | 0.24 |  |
| **rs6463266** | A/A |  | 684 | 27.0 | 27.0 |  |
|  | A/C |  | 362 | 26.3 | 26.2 |  |
|  | C/C |  | 43 | 28.3 | 28.3 |  |
| P-trend ^3^ |  |  |  | 0.96 | 0.93 |  |
| **rs3852789** | T/T |  | 694 | 26.5 | 26.4 |  |
|  | T/G |  | 391 | 27.5 | 27.7 |  |
|  | G/G |  | 38 | 28.8 | 27.3 |  |
| P-trend ^3^ |  |  |  | 0.43 | 0.36 |  |
| **rs12658840** | G/G |  | 525 | 26.4 | 26.4 |  |
|  | A/G |  | 483 | 27.6 | 27.8 |  |
|  | A/A |  | 116 | 27.8 | 26.7 |  |
| P-trend ^3^ |  |  |  | 0.21 | 0.32 |  |
| **rs2298075** | G/G |  | 668 | 28.2 | **27.6** |  |
|  | G/T |  | 374 | 25.8 | **26.5** |  |
|  | T/T |  | 55 | 21.9 | **23.2** |  |
| P-trend ^3^ |  |  |  | 0.004 | **0.05** |  |
| **rs7307700** | G/G |  | 317 | 28.1 | 27.4 |  |
|  | A/G |  | 558 | 27.0 | 27.2 |  |
|  | A/A |  | 260 | 25.0 | 25.2 |  |
| P-trend ^3^ |  |  |  | 0.07 | 0.16 |  |

1 Age adjusted

^2^ Multivariate adjusted for the following: age (continuous), body mass index (BMI) (continuous), alcohol consumption (none, <5 g/day, 5-14.9 g/day, 15+ g/day), age at first birth/parity (nulliparous, age at first birth <25, age at first birth 25-29, age at first birth 30+), history of benign breast disease (yes/no), family history of breast cancer (yes/no).

^3^ Multivariate adjusted for the following: age, BMI, alcohol consumption, age at first birth/parity, history of benign breast disease, family history of breast cancer, postmenopausal status/hormone use (premenopausal, never user, current user, past user).

^4^ P-trend based on genotype coded as ordinal variable regressed on square root transformed MD.
