# Supplementary material for: Breast cancer susceptibility loci and mammographic density
Source: Breast Cancer Res. 2008 Aug 5;10(4):R66. doi: 10.1186/bcr2127 (PMC2575539; doi:10.1186/bcr2127)
Supplement: Additional file 2 — A Word document containing a table that lists the mean absolute (cm2) dense area according to breast cancer susceptibility loci, Nurses' Health Study controls (1989 to 1998). [file bcr2127-S2.doc]

Supplementary Table 2: Mean absolute (cm^2^) dense area according to breast cancer susceptibility loci, Nurses’ Health Study controls (1989-1998)

|  |  | Premenopausal (n=217) | | | |  | Postmenopausal (n=904) | | |
| --- | --- | --- | --- | --- | --- | --- | --- | --- | --- |
|  |  |  | N | Mean MD^1^ | Mean MD^2^ |  | N | Mean MD^1^ | Mean MD^3^ |
| **rs2981582** | G/G |  | 73 | 42.6 | 42.8 |  | 332 | 29.0 | 28.8 |
|  | G/A |  | 112 | 44.0 | 44.3 |  | 407 | 28.8 | 28.6 |
|  | A/A |  | 28 | 43.3 | 41.8 |  | 131 | 28.2 | 29.2 |
| P-trend ^4^ |  |  |  | 0.66 | 0.89 |  |  | 0.40 | 0.66 |
| **rs12443621** | A/A |  | 51 | 39.8 | **40.2** |  | 236 | 27.4 | 27.9 |
|  | A/G |  | 104 | 40.6 | **40.7** |  | 442 | 29.3 | 29.0 |
|  | G/G |  | 54 | 51.3 | **50.8** |  | 202 | 28.4 | 28.4 |
| P-trend ^4^ |  |  |  | 0.03 | **0.04** |  |  | 0.61 | 0.75 |
| **rs13281615** | A/A |  | 70 | 42.9 | 42.7 |  | 290 | 30.6 | 30.4 |
|  | A/G |  | 94 | 39.2 | 39.6 |  | 445 | 28.5 | 28.6 |
|  | G/G |  | 47 | 51.4 | 51.0 |  | 144 | 25.7 | 25.7 |
| P-trend ^4^ |  |  |  | 0.25 | 0.29 |  |  | 0.11 | 0.11 |
| **rs3817198** | A/A |  | 80 | 38.0 | **37.7** |  | 396 | 27.7 | 27.6 |
|  | A/G |  | 106 | 45.2 | **45.3** |  | 368 | 29.7 | 29.8 |
|  | G/G |  | 20 | 46.1 | **47.7** |  | 93 | 29.0 | 29.1 |
| P-trend ^4^ |  |  |  | 0.06 | **0.04** |  |  | 0.82 | 0.67 |
| **rs889312** | T/T |  | 107 | 48.3 | 44.3 |  | 499 | 29.7 | 29.6 |
|  | T/G |  | 89 | 42.0 | 42.0 |  | 321 | 26.7 | 26.9 |
|  | G/G |  | 14 | 44.0 | 46.7 |  | 58 | 32.0 | 31.6 |
| P-trend ^4^ |  |  |  | 0.23 | 0.50 |  |  | 0.39 | 0.40 |
| **rs4666451** | G/G |  | 74 | 45.6 | 45.7 |  | 314 | 29.8 | 30.0 |
|  | A/G |  | 96 | 43.9 | 43.9 |  | 418 | 28.7 | 28.4 |
|  | A/A |  | 36 | 38.4 | 38.4 |  | 144 | 26.8 | 27.4 |
| P-trend ^4^ |  |  |  | 0.09 | 0.22 |  |  | 0.19 | 0.12 |
| **rs2107425** | G/G |  | 105 | 47.0 | 47.2 |  | 449 | 29.9 | 30.3 |
|  | G/A |  | 87 | 40.1 | 39.9 |  | 337 | 27.3 | 26.9 |
|  | A/A |  | 18 | 37.5 | 38.4 |  | 85 | 28.2 | 27.6 |
| P-trend ^4^ |  |  |  | 0.09 | 0.10 |  |  | 0.28 | 0.11 |
| **rs981782** | A/A |  | 64 | 38.5 | 38.1 |  | 258 | 29.9 | 29.5 |
|  | A/C |  | 100 | 45.0 | 45.3 |  | 429 | 29.0 | 29.3 |
|  | C/C |  | 47 | 46.9 | 47.1 |  | 175 | 27.2 | 27.1 |
| P-trend ^4^ |  |  |  | 0.20 | 0.17 |  |  | 0.13 | 0.19 |
| **rs8051542** | G/G |  | 63 | 45.6 | 45.7 |  | 234 | 28.0 | 28.1 |
|  | G/A |  | 73 | 43.8 | 43.6 |  | 296 | 26.7 | 27.0 |
|  | A/A |  | 32 | 40.7 | 41.4 |  | 137 | 31.9 | 31.1 |
| P-trend ^4^ |  |  |  | 0.67 | 0.72 |  |  | 0.57 | 0.73 |
| **rs30099** | C/C |  | 175 | 41.9 | 42.0 |  | 723 | 29.3 | 29.3 |
|  | C/T |  | 34 | 46.6 | 46.5 |  | 151 | 25.9 | 25.7 |
|  | T/T |  | 0 | - |  |  | 8 | 25.3 | 22.8 |
| P-trend ^4^ |  |  |  | 0.28 | 0.29 |  |  | 0.22 | 0.11 |
| **rs3803662** | G/G |  | 107 | 40.8 | 40.8 |  | 458 | 28.4 | 28.6 |
|  | G/A |  | 77 | 45.7 | 45.4 |  | 344 | 28.7 | 28.3 |
|  | A/A |  | 20 | 40.6 | 41.7 |  | 69 | 31.5 | 32.3 |
| **P-trend ^4^** |  |  |  | 0.43 | 0.41 |  |  | 0.55 | 0.57 |

1 Age adjusted

^2^ Multivariate adjusted for the following: age (continuous), body mass index (BMI) (continuous), alcohol consumption (none, <5 g/day, 5-14.9 g/day, 15+ g/day), age at first birth/parity (nulliparous, age at first birth <25, age at first birth 25-29, age at first birth 30+), history of benign breast disease (yes/no), family history of breast cancer (yes/no).

^3^ Multivariate adjusted for the following: age, BMI, alcohol consumption, age at first birth/parity, history of benign breast disease, family history of breast cancer, postmenopausal hormone use (never user, current user, past user).

^4^ P-trend based on genotype coded as ordinal variable regressed on square root transformed MD.
