# Supplementary material for: Breast cancer susceptibility loci and mammographic density
Source: Breast Cancer Res. 2008 Aug 5;10(4):R66. doi: 10.1186/bcr2127 (PMC2575539; doi:10.1186/bcr2127)
Supplement: Additional file 3 — A Word document containing a table that lists the mean absolute (cm2) non-dense area according to breast cancer susceptibility loci, Nurses' Health Study controls (1989 to 1998). [file bcr2127-S3.doc]

Supplementary Table 3: Mean absolute (cm^2^) nondense area according to breast cancer susceptibility loci, Nurses’ Health Study controls (1989-1998)

|  |  | Premenopausal (n=217) | | | |  | Postmenopausal (n=904) | | |
| --- | --- | --- | --- | --- | --- | --- | --- | --- | --- |
|  |  |  | N | Mean MD^1^ | Mean MD^2^ |  | N | Mean MD^1^ | Mean MD^3^ |
| **rs2981582** | G/G |  | 73 | 80.3 | 78.7 |  | 332 | 108.8 | 109.6 |
|  | G/A |  | 112 | 74.4 | 73.5 |  | 407 | 107.8 | 109.4 |
|  | A/A |  | 28 | 66.7 | 76.4 |  | 131 | 114.7 | 107.7 |
| P-trend ^4^ |  |  |  | 0.19 | 0.55 |  |  | 0.54 | 0.65 |
| **rs12443621** | A/A |  | 51 | 84.1 | 81.5 |  | 236 | 113.5 | 113.1 |
|  | A/G |  | 104 | 73.8 | 74.9 |  | 442 | 107.9 | 109.1 |
|  | G/G |  | 54 | 70.8 | 72.4 |  | 202 | 109.2 | 107.1 |
| P-trend ^4^ |  |  |  | 0.25 | 0.30 |  |  | 0.55 | 0.27 |
| **rs13281615** | A/A |  | 70 | 69.1 | 73.9 |  | 290 | 110.3 | 110.3 |
|  | A/G |  | 94 | 82.3 | 75.3 |  | 445 | 110.2 | 109.7 |
|  | G/G |  | 47 | 71.2 | 79.3 |  | 144 | 103.5 | 105.2 |
| P-trend ^4^ |  |  |  | 0.92 | 0.91 |  |  | 0.49 | 0.43 |
| **rs3817198** | A/A |  | 80 | 82.9 | 81.5 |  | 396 | 105.2 | 106.4 |
|  | A/G |  | 106 | 70.1 | 71.5 |  | 368 | 110.8 | 109.8 |
|  | G/G |  | 20 | 81.3 | 81.7 |  | 93 | 117.4 | 116.3 |
| P-trend ^4^ |  |  |  | 0.23 | 0.15 |  |  | 0.08 | 0.06 |
| **rs889312** | T/T |  | 107 | 74.7 | 74.3 |  | 499 | 106.3 | 107.7 |
|  | T/G |  | 89 | 76.1 | 76.2 |  | 321 | 113.9 | 110.3 |
|  | G/G |  | 14 | 67.4 | 74.0 |  | 58 | 98.6 | 106.6 |
| P-trend ^4^ |  |  |  | 0.92 | 0.77 |  |  | 0.47 | 0.45 |
| **rs4666451** | G/G |  | 74 | 73.6 | **71.6** |  | 314 | 112.6 | 109.2 |
|  | A/G |  | 96 | 72.5 | **75.8** |  | 418 | 107.6 | 109.3 |
|  | A/A |  | 36 | 93.5 | **90.4** |  | 144 | 104.8 | 107.0 |
| P-trend ^4^ |  |  |  | 0.13 | **0.02** |  |  | 0.13 | 0.46 |
| **rs2107425** | G/G |  | 105 | 70.2 | 74.3 |  | 449 | 110.9 | 110.7 |
|  | G/A |  | 87 | 80.7 | 78.2 |  | 337 | 108.8 | 108.0 |
|  | A/A |  | 18 | 87.5 | 79.4 |  | 85 | 104.4 | 108.8 |
| P-trend ^4^ |  |  |  | 0.18 | 0.77 |  |  | 0.38 | 0.52 |
| **rs981782** | A/A |  | 64 | 77.4 | 76.7 |  | 258 | 103.9 | 108.4 |
|  | A/C |  | 100 | 74.7 | 75.9 |  | 429 | 110.2 | 108.4 |
|  | C/C |  | 47 | 74.9 | 74.7 |  | 175 | 112.1 | 109.9 |
| P-trend ^4^ |  |  |  | 0.75 | 0.69 |  |  | 0.14 | 0.70 |
| **rs8051542** | G/G |  | 63 | 70.2 | 72.2 |  | 234 | 110.1 | 109.0 |
|  | G/A |  | 73 | 71.7 | 71.1 |  | 296 | 113.3 | 114.4 |
|  | A/A |  | 32 | 81.0 | 80.3 |  | 137 | 107.5 | 107.1 |
| P-trend ^4^ |  |  |  | 0.35 | 0.37 |  |  | 0.51 | 0.50 |
| **rs30099** | C/C |  | 175 | 77.6 | 77.5 |  | 723 | 109.1 | 108.6 |
|  | C/T |  | 34 | 68.8 | 71.5 |  | 151 | 109.9 | 111.1 |
|  | T/T |  | 0 |  |  |  | 8 | 97.9 | 115.8 |
| P-trend ^4^ |  |  |  | 0.64 | 0.82 |  |  | 0.96 | 0.43 |
| **rs3803662** | G/G |  | 107 | 76.8 | 76.7 |  | 458 | 108.5 | 108.3 |
|  | G/A |  | 77 | 71.5 | 74.9 |  | 344 | 109.5 | 111.5 |
|  | A/A |  | 20 | 83.3 | 74.3 |  | 69 | 109.8 | 101.6 |
| **P-trend ^4^** |  |  |  | 0.96 | 0.66 |  |  | 0.97 | 0.59 |

1 Age adjusted

^2^ Multivariate adjusted for the following: age (continuous), body mass index (BMI) (continuous), alcohol consumption (none, <5 g/day, 5-14.9 g/day, 15+ g/day), age at first birth/parity (nulliparous, age at first birth <25, age at first birth 25-29, age at first birth 30+), history of benign breast disease (yes/no), family history of breast cancer (yes/no).

^3^ Multivariate adjusted for the following: age, BMI, alcohol consumption, age at first birth/parity, history of benign breast disease, family history of breast cancer, postmenopausal hormone use (never user, current user, past user).

^4^ P-trend based on genotype coded as ordinal variable regressed on square root transformed MD.
