# Supplementary material for: Breast cancer susceptibility loci and mammographic density
Source: Breast Cancer Res. 2008 Aug 5;10(4):R66. doi: 10.1186/bcr2127 (PMC2575539; doi:10.1186/bcr2127)
Supplement: Additional file 4 — A Word document containing a table that lists the mean percentage mammographic density according to second-stage SNPs that were not validated in stage 3 BCAC SNPs among controls. [file bcr2127-S4.doc]

Supplemental Table 4: Mean percent mammographic density according to 2^nd^ stage SNPs that were not validated in Stage 3 BCAC SNPs among controls

|  |  | Premenopausal (N=217) | | |  | Postmenopausal (N=904) | | |  |
| --- | --- | --- | --- | --- | --- | --- | --- | --- | --- |
|  |  | N | Mean %MD^1^ | Mean %MD^2^ |  | N | Mean %MD^1^ | Mean %MD^2^ |  |
| **rs3857481** | G/G | 102 | 37.0 | 38.1 |  | 406 | 23.4 | 23.4 |  |
|  | A/G | 88 | 42.7 | 42.6 |  | 392 | 24.4 | 24.6 |  |
|  | A/A | 22 | 35.8 | 36.4 |  | 80 | 23.4 | 22.6 |  |
| P-trend ^3^ |  |  | 0.47 | 0.20 |  |  | 0.88 | 0.92 |  |
| **rs1318703** | A/A | 71 | 37.6 | 37.1 |  | 302 | 24.3 | 24.7 |  |
|  | A/G | 99 | 39.3 | 39.5 |  | 423 | 23.5 | 23.4 |  |
|  | G/G | 41 | 41.6 | 41.2 |  | 140 | 23.7 | 23.1 |  |
| P-trend ^3^ |  |  | 0.25 | 0.16 |  |  | 0.72 | 0.27 |  |
| **rs16998733** | G/G | 164 | 40.1 | 39.7 |  | 692 | 23.9 | 23.9 |  |
|  | A/G | 40 | 36.4 | 36.8 |  | 168 | 26.2 | 26.0 |  |
|  | A/A | 6 | 33.0 | 35.9 |  | 14 | 16.0 | 17.8 |  |
| P-trend ^3^ |  |  | 0.19 | 0.32 |  |  | 0.35 | 0.22 |  |
| **rs2314099** | C/C | 189 | 39.1 | 39.2 |  | 774 | 24.2 | 24.2 |  |
|  | C/A | 22 | 41.3 | 39.6 |  | 96 | 22.4 | 22.6 |  |
|  | A/A | 0 | - | - |  | 6 | 24.4 | 30.5 |  |
| P-trend ^3^ |  |  | 0.59 | 0.84 |  |  | 0.38 | 0.60 |  |
| **rs4841365** | G/G | 105 | 40.4 | 39.9 |  | 422 | 24.6 | 24.4 |  |
|  | G/C | 78 | 39.3 | 39.4 |  | 357 | 23.7 | 24.0 |  |
|  | C/C | 20 | 33.0 | 34.2 |  | 82 | 25.4 | 24.9 |  |
| P-trend ^3^ |  |  | 0.37 | 0.55 |  |  | 0.92 | 0.98 |  |
| **rs7313833** | G/G | 100 | 39.8 | 40.5 |  | 380 | 23.9 | 23.8 |  |
|  | A/G | 90 | 39.7 | 38.5 |  | 383 | 24.1 | 24.4 |  |
|  | A/A | 21 | 38.3 | 39.0 |  | 107 | 26.3 | 25.9 |  |
| P-trend ^3^ |  |  | 0.74 | 0.56 |  |  | 0.44 | 0.34 |  |
| **rs11235127** | G/G | 133 | 37.5 | 37.0 |  | 596 | 24.7 | 24.6 |  |
|  | A/G | 65 | 41.5 | 43.0 |  | 240 | 23.4 | 23.5 |  |
|  | A/A | 8 | 40.1 | 33.7 |  | 32 | 21.2 | 23.5 |  |
| P-trend ^3^ |  |  | 0.26 | 0.19 |  |  | 0.34 | 0.70 |  |
| **rs17157070** | A/A | 196 | 39.9 | 39.4 |  | 802 | 24.4 | 24.1 |  |
|  | A/G | 14 | 35.7 | 40.0 |  | 76 | 22.3 | 25.2 |  |
|  | G/G | 2 | 4.1 | 13.9 |  | 1 | 0.0 | 10.9 |  |
| P-trend ^3^ |  |  | 0.04 | 0.20 |  |  | 0.11 | 0.99 |  |
| **rs6469633** | A/A | 109 | 38.6 | 39.4 |  | 504 | 22.9 | **22.9** |  |
|  | A/G | 90 | 39.4 | 37.8 |  | 323 | 25.9 | **26.0** |  |
|  | G/G | 8 | 35.6 | 38.7 |  | 45 | 26.1 | **26.2** |  |
| P-trend ^3^ |  |  | 0.90 | 0.53 |  |  | 0.02 | **0.008** |  |
| **rs4331913** | G/G | 73 | 37.2 | 37.4 |  | 291 | 24.4 | 24.2 |  |
|  | G/A | 102 | 42.1 | 42.4 |  | 434 | 23.6 | 23.9 |  |
|  | A/A | 35 | 34.6 | 32.9 |  | 151 | 24.4 | 24.1 |  |
| P-trend ^3^ |  |  | 0.78 | 0.53 |  |  | 0.43 | 0.32 |  |
| **rs6843340** | G/G | 66 | 34.8 | **34.2** |  | 234 | 22.1 | 22.0 |  |
|  | G/A | 99 | 42.0 | **41.3** |  | 432 | 25.0 | 25.1 |  |
|  | A/A | 46 | 40.0 | **41.6** |  | 213 | 25.0 | 24.9 |  |
| P-trend ^3^ |  |  | 0.06 | **0.009** |  |  | 0.10 | 0.05 |  |
| **rs2049621** | C/C | 173 | 38.4 | 38.5 |  | 719 | 24.2 | 24.5 |  |
|  | C/G | 35 | 42.1 | 41.6 |  | 150 | 23.7 | 22.5 |  |
|  | G/G | 2 | 49.6 | 38.5 |  | 9 | 28.9 | 29.3 |  |
| P-trend ^3^ |  |  | 0.28 | 0.50 |  |  | 0.89 | 0.49 |  |
| **Rs10508468** | A/A | 96 | 36.5 | 37.5 |  | 343 | 24.1 | 23.3 |  |
|  | A/G | 84 | 39.9 | 38.9 |  | 411 | 24.3 | 25.1 |  |
|  | G/G | 31 | 46.7 | 45.6 |  | 117 | 22.7 | 22.2 |  |
| P-trend ^3^ |  |  | 0.03 | 0.09 |  |  | 0.84 | 0.66 |  |
| **rs13110927** | G/G | 58 | 35.5 | 37.3 |  | 218 | 24.3 | 24.8 |  |
|  | G/A | 98 | 43.1 | 41.3 |  | 456 | 24.0 | 23.7 |  |
|  | A/A | 53 | 34.9 | 35.7 |  | 201 | 23.7 | 24.1 |  |
| P-trend ^3^ |  |  | 0.94 | 0.67 |  |  | 0.87 | 0.83 |  |
| **rs4954956** | G/G | 119 | 39.7 | 38.4 |  | 478 | 23.9 | 24.0 |  |
|  | G/A | 81 | 40.5 | 40.4 |  | 342 | 23.9 | 24.0 |  |
|  | A/A | 13 | 28.7 | 39.8 |  | 61 | 28.2 | 27.3 |  |
| P-trend ^3^ |  |  | 0.43 | 0.43 |  |  | 0.34 | 0.36 |  |
| **rs6463266** | A/A | 130 | 38.4 | 38.8 |  | 527 | 24.5 | 24.4 |  |
|  | A/C | 65 | 38.8 | 37.7 |  | 288 | 23.4 | 23.5 |  |
|  | C/C | 10 | 45.3 | 44.6 |  | 33 | 23.9 | 23.7 |  |
| P-trend ^3^ |  |  | 0.59 | 0.87 |  |  | 0.70 | 0.68 |  |
| **rs3852789** | T/T | 138 | 38.1 | 38.4 |  | 529 | 23.9 | 23.8 |  |
|  | T/G | 68 | 41.8 | 40.8 |  | 313 | 24.4 | 24.7 |  |
|  | G/G | 2 | 36.6 | 38.2 |  | 34 | 27.1 | 26.0 |  |
| P-trend ^3^ |  |  | 0.38 | 0.57 |  |  | 0.51 | 0.31 |  |
| **rs12658840** | G/G | 102 | 37.0 | 37.5 |  | 406 | 24.2 | 24.1 |  |
|  | A/G | 85 | 43.2 | 42.4 |  | 383 | 24.2 | 24.5 |  |
|  | A/A | 26 | 37.6 | 36.8 |  | 84 | 24.9 | 24.0 |  |
| P-trend ^3^ |  |  | 0.24 | 0.46 |  |  | 0.68 | 0.69 |  |
| **rs2298075** | G/G | 126 | 39.8 | 39.1 |  | 522 | 25.7 | **25.1** |  |
|  | G/T | 74 | 37.9 | 39.0 |  | 286 | 22.9 | **23.8** |  |
|  | T/T | 9 | 46.4 | 44.3 |  | 44 | 16.7 | **18.7** |  |
| P-trend ^3^ |  |  | 0.88 | 0.54 |  |  | 0.0009 | **0.02** |  |
| **rs7307700** | G/G | 62 | 41.0 | 40.0 |  | 247 | 25.2 | 24.5 |  |
|  | A/G | 104 | 40.5 | 40.5 |  | 434 | 24.0 | 24.3 |  |
|  | A/A | 45 | 35.1 | 35.8 |  | 205 | 22.7 | 23.0 |  |
| P-trend ^3^ |  |  | 0.20 | 0.39 |  |  | 0.17 | 0.35 |  |

1 Age adjusted

^2^ Multivariate adjusted for the following: age (continuous), body mass index (BMI) (continuous), alcohol consumption (none, <5 g/day, 5-14.9 g/day, 15+ g/day), age at first birth/parity (nulliparous, age at first birth <25, age at first birth 25-29, age at first birth 30+), history of benign breast disease (yes/no), family history of breast cancer (yes/no).

^3^ Multivariate adjusted for the following: age, BMI, alcohol consumption, age at first birth/parity, history of benign breast disease, family history of breast cancer, postmenopausal hormone use (never user, current user, past user).

^4^ P-trend based on genotype coded as ordinal variable regressed on square root transformed MD.
